# A facile ionic-liquid pretreatment method for the examination of archaeological wood by scanning electron microscopy

**DOI:** 10.1038/s41598-019-49773-y

**Published:** 2019-09-13

**Authors:** Bing-Jyun Lu, Jia-Rong Li, Hwan-Ching Tai, Wenjie Cai, Hsiao-Han Tseng, Yi-Ting Hsieh

**Affiliations:** 1 Department of Chemistry, Soochow University, Taipei City, 11102 Taiwan; 2 Department of Chemistry, National Taiwan University, Taipei, 106 Taiwan; 3 College of Arts and Landscape Architecture, Fujian Agriculture and Forestry University, Fuzhou, 350002 China; 4 Graduate Institute of Creative Industries, College of Management, Shih Chien University, Taipei, 104 Taiwan

**Keywords:** Characterization and analytical techniques, Imaging studies

## Abstract

Wood has been a crucial natural material for human civilization since prehistoric times. In archaeology, the examination of the wood microstructure is important for the study of architecture, musical instruments, sculptures, and so on. Scanning electron microscopy (SEM) examination is sometimes unsuitable for archaeological wood due to the limited amount of precious samples, which may be too small to be cut by microtomes and mounted on holders. Moreover, the conductive coating material cannot be uniformly deposited over uneven wood surfaces. To overcome these issues, a rapid and simple pretreatment method using room-temperature ionic liquids (RTIL) was proposed. Four common RTILs were evaluated for the pretreatment of wood chips for SEM examination. We found that water content, viscosity, density, and hydrophobicity of IL solutions were important factors affecting SEM image quality. A 7.5% solution of 1-butyl-1-methylpyrrolidium dicyanamide (BMP-DCA) in ethanol (v/v) was found to work very well. The IL pretreatment could be performed in a few minutes without special equipment. It is gentle enough to preserve delicate structures such as the torus/margo of pit membranes, even at elevated temperatures, without causing obvious damage or deformation. We successfully imaged hand-cut wood chips from 18^th^-century buildings, an 18^th^-century European violin, and a Chinese zither over 1000 years old. We therefore conclude that highly hydrophilic ionic liquids with low density and viscosity are suitable for use in SEM examinations of both modern and antique wood specimens.

## Introduction

Scanning electron microscopy (SEM) is a vital technique for mapping out the morphologies and the structures of various materials. SEM systems are usually operated within a high vacuum chamber, and the samples are typically coated with a conductive layer; thus, the specimen must be nonvolatile and conductive. Because of these restrictions, wet and liquid samples cannot be directly introduced into the chamber. To overcome these issues, conventional pretreatments used for the biological specimens prior to an SEM examination includes the following processes: chemical fixation, dehydration, freezing critical point drying, followed by applying a conductive film coating^1^.

Because conventional pretreatments are sophisticated and time-consuming, many techniques have been developed to observe various biomaterials and wet samples, including cryo-SEM and environmental SEM. Although these techniques provide useful images for biomaterial samples in their hydrated states, the facilities are rather expensive^2^. An alternative is to consider the application of room-temperature ionic liquids (RTILs) in SEM experiments. RTILs are a type of liquid salts that are ionic in nature. RTILs have many unique properties compared to conventional solvents, in that they have good thermal stability, a high ionic conductivity, a wide electrochemical window and negligible vapor pressure. These unique properties have enabled ILs to be applied to many areas of synthesis, separation, batteries, and electrodeposition^3–7^. Due to their negligible vapor pressure, ionic liquids can be used under conditions of high vacuum. A number of studies have reported on the use of ILs in SEM^8–15^. Kuwabata *et al*., first reported on the use of hydrophobic 1-ethyl-3-methylimidazolium bis(trifluoromethylsulfony)imide (EMI-TFSI) ILs as an electronically conducting material on an insulating star stand for SEM observation in order to prevent the accumulation of electron charges^16^. Ionic liquids can be used as a pretreatment reagent not only for insulating samples but also for the wet specimens and biological materials. Tsuda *et al*. obtained very clearly SEM images of hydrous superabsorbent polymer particles using the tri-n-butylmethylphosphonium dimethylphosphate IL as a pretreatment reagent^15^. The water molecules that intercalated in the hydrated montmorillonite can be replaced by 1-butyl-3-methylimidazolium tetrafluoroborate IL^12^. Hyno *et al*. reported the structure and size of human blood cells could be maintained without shrinkage when they are pretreated with a hydrophilic, choline-like IL^17^. These studies have shown that hydrophilic ILs can penetrate into a wet specimen and replace the water molecules therein.

Understanding the nature and characteristics of wood is important in many industrial and academic research projects, relating to the pulp industry, biomass conversion, architecture, studies of cultural heritage, etc. An interesting example is the examination of wood shavings removed from Stradivarius violins during repairs to uncover the “lost secrets” of Antonio Stradivari, the most successful violin maker of all time. Nagyvary *et al*.^18^ have reported that Stradivari’s spruce showed fungal growth and bacterial next to partially degraded pit membranes. They suggested that Stradivari’s spruce was once floated down rivers or stored in ponds. However, Barlow *et al*.^19^ also examined Stradivari’s spruce by SEM but only found intact pit membranes without traces of bacteria or fungi. This dispute remains unresolved to this date because it has been very difficult to obtain sufficient amounts of Stradivari’s wood for follow-up studies. Conventionally, prior to the use of SEM for such examinations, wood samples needed to be surfaced with a sliding microtome, mounted on a holder and then coated with a conductive layer. However, for certain precious wood samples, this conventional method cannot be applied due to the limited amount of sample available.

Yamasita *et al*. obtained acceptable SEM images by using 1-ethyl-3-methylimidazorlium methylphosphonate IL as a pretreatment reagent for observing wood specimens (keyaki and hinoki) in SEM^20^. The image quality appeared to be inferior compared to conventional graphite/metal coatings, and only a specific IL was tested. We hypothesize that further improvements in SEM imaging quality could be achieved if we systematically compare multiple ILs and explore associated parameters.

In this study, we selected four commonly used RTILs with different degrees of hydrophilicity for SEM examination because the IL coverage and the wettability of the target samples would influence the conductivity. We investigated the effects of different IL and water concentrations on the quality of SEM and the elemental analysis by energy-dispersive X-ray spectroscopy (EDX). We found an optimal formulation for IL pretreatment of wood, and the treated sample could even be imaged at elevated temperatures. This study not only examined modern spruce wood but also focused on precious antique wood samples obtained from architectural sources and musical instruments. The antique wood samples were tiny pieces cut by hand, with uneven surfaces, and too small to be mounted on a holder; nonetheless, they could be effectively imaged after IL treatment. Our proposed method is generally useful in wood science research because of reduced sample preparation time and increased throughput, and particularly suitable for archeological studies and other applications for which only very small wood samples are available.

## Results and Discussion

A thin and uniform conducting layer forming on the target samples for SEM observation is important. The hydrophilicity of IL and the wettability of woods may alter the properties of this layer. Thus, four types of ILs are selected for comparison-

**Figure 1:**
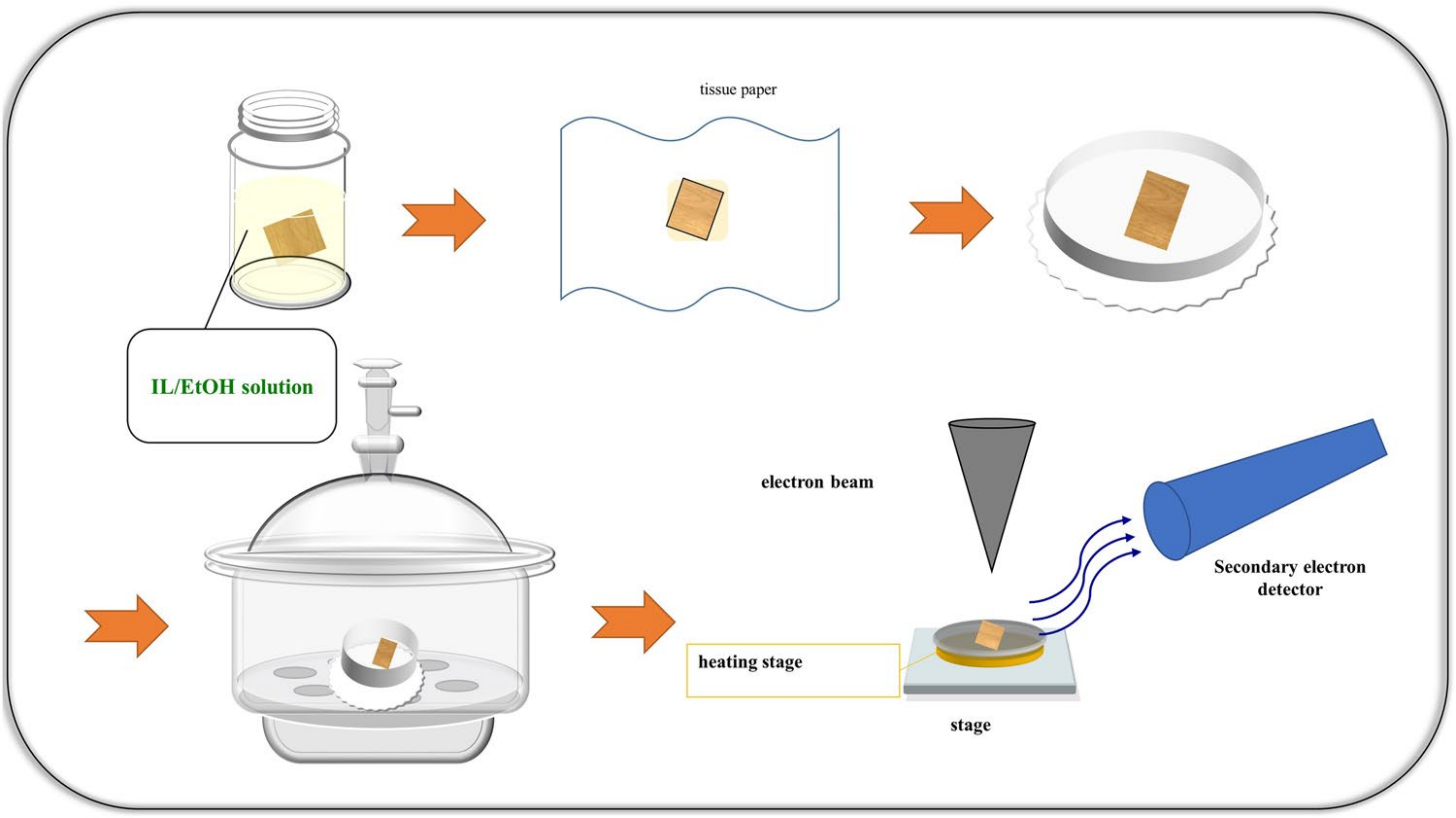
Illustration of the pretreatment protocol for spruce samples for SEM observations.

1-ethyl-3-methylimidazolium dicyanamide (EMI-DCA), 1-ethyl-3-methylimidazolium bis(trifluoromethylsulfony)imide (EMI-TFSI), 1- butyl-1- methylpyrrolidium dicyanamide (BMP-DCA), 1- butyl-1- methylpyrrolidium bis(trifluoromethylsulfony)imide (BMP-TFSI) and their structures are shown in Fig. S1. Modern spruce (*Picea abies*) specimens were immersed in the different ILs using the pretreatment process described in Fig. 1. As opposed to the conventional method, none of the samples needed to be cut with a sliding microtome nor to be mounted in a stub during this process. All samples were observed at the same acceleration voltage of 5 kV. To understand the influence of the IL/EtOH solution on the quality of SEM images, physical properties such as water content, viscosity, and density were determined, and the results are shown in Table 1.

**Table 1:** Physicochemical properties of the ILs at 20 °C.

	IL/EtOH	Water content (ppm)	Viscosity (cP)	Density (gcm^−3^)
EMI-TFSI	neat	25.6	55.9	1.54
	5%	4.50 * 10^4^	—	0.843
EMI-DCA	neat	33.7	20.8	1.11
	5%	4.90 * 10^4^	—	0.825
BMP-TFSI	neat	15.4	307	1.46
	10%	3.86 * 10^4^	—	0.862
BMP-DCA	neat	31.3	49.6	1.02
	7.5%	4.62 * 10^4^	—	0.825

**Figure 2:**
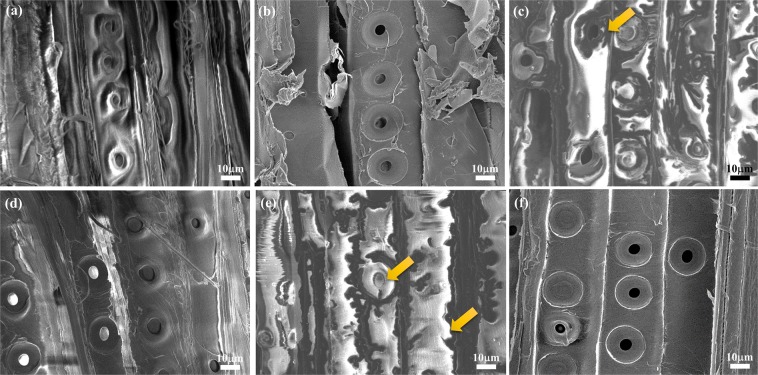
SEM images of modern spruce pretreated with (**a**) no treatment, (**b**) platinum sputtering, (**c**) 5% EMI-TFSI, (**d**) 5% EMI-DCA, (**e**) 10% BMP-TFSI, and (**f**) 7.5% BMP-DCA/ethanol solutions. The acceleration voltage is 5 kV.

The insulating modern spruce wood sample in Fig. 2(a) is given a white SEM image due to the accumulation of electron charges, and the sample is damaged during the observation. Then, the wood sample was treated by a conventional metal-sputtering method and the SEM image in Fig. 2(b) presents a clear image as expected. However, the high surface roughness of the sample sometimes causes the electrostatic charging on some areas. Taking advantage of the ILs behaving as electrically conducting materials, modern spruce specimens which were immersed in four kinds of ILs dissolved in ethanol (in their optimal concentration) for SEM examinations in Fig. 2(c–f). The hydrophobic ILs, 5% EMI-TFSI and 10% BMP-TFSI, only yielded poor-quality images like those in Fig. 2(c,e), respectively. The white area around the pits of the wood indicates that serious electrostatic charging had developed at the surface and the IL had accumulated at the edge (indicated by arrows). Based on the water content and viscosity data shown in Table 1, the TFSI-based ILs had a higher viscosity and a lower water content compared to the DCA-based ILs. The results indicate that ILs with a higher viscosity and lower water content, such as BMP-TFSI, cannot spread well on the samples. In contrast, as shown in Fig. 2(d,f), spruce specimens pretreated with hydrophilic ILs with 5% EMI-DCA and 7.5% BMP-DCA could be imaged at better quality. In fact, BMP-DCA/ethanol solution provided the best SEM image quality, comparable to the conventional metal coating. Both allowed the clear visualization of the torus/margo structure of pit membranes under high SEM magnification (Fig. S2). It suggests that a very thin and well dispersed IL layer coated the sample surface and it worked effectively as an antistatic agent under the electron beam.

According to previous studies^15^, ILs with relatively low densities would be expected to generate better SEM images due to the fact that the depth of penetration of the primary electron beam is inversely proportional to the density of the target materials^21^. Table 1 shows the density of the four ILs used in this study-BMP-DCA and EMI-DCA have lower densities than EMI-TFSI and BMP-TFSI. Because the samples pretreated with BMP-DCA showed a much better SEM image quality, we used BMP-DCA as the pretreatment reagent for the following studies.

**Figure 3:**
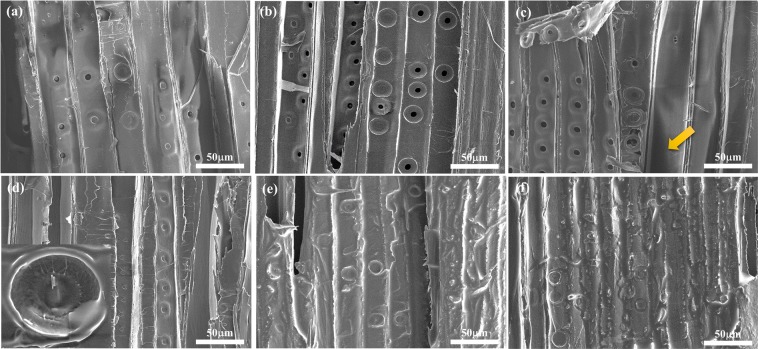
SEM images of modern spruce pretreated with (**a**) 5, (**b**) 7.5, (**c**) 10, (**d**) 25, (**e**) 50, and (**f**) 100% of BMP-DCA/ ethanol solutions. The acceleration voltage is 5 kV.

To acquire optimal SEM images, the conducting layer over the sample should be thin and uniform. Hence, too much or too little IL coating could compromise image quality. Thus, we carried out tests to determine the optimal concentration of BMP-DCA solution, using radially cut spruce samples, shown in Fig. 3. At 5%, the image in Fig. 3(a) was blurred with electrostatic charging on the surface. There was probably too little IL to cover the entire wood surface. Better images were obtained at 7.5% (Fig. 3b) and 10% (Fig. 3c), but the latter showed excessive accumulation of IL at some places (yellow arrow). At concentrations above 25% (Fig. 3d–f), the images became dark and blurred. Excessive IL formed a thick layer that prevented fine structures such as pit membranes to be clearly resolved (Fig. 3d inset). Consequently, we concluded that the 7.5% BMP-DCA solution was optimum, producing a well-spread and thin conductive layer. Our results are consistent with those of a previous study^20^, in which 10% EMI-MePO_3_Me was found to be optimal for imaging Japanese cedar-having a higher or a lower concentration both yielded poorer SEM images. A number of studies indicated that some ionic liquids might change the morphology and chemical composition of wood tissues^19,22,23^. In such cases, the pit membrane in bordered pits became broken as the cellulose was solubilized by IL treatment^19^. To ensure that BMP-DCA does cause similar damages, we carried out experiments in which the wood samples were immersed in both neat BMP-DCA and 7.5% BMP-DCA solutions for one month. The spruce pit membranes remained intact in both cases. In addition, a single wood sample was treated four times, cleaned with deionized water and examined by SEM after each treatment. No differences were found after repeated treatments. Thus, we chose the 7.5% BMP-DCA solution as the pretreatment reagent for the study of archaeological wood.

**Figure 4:**
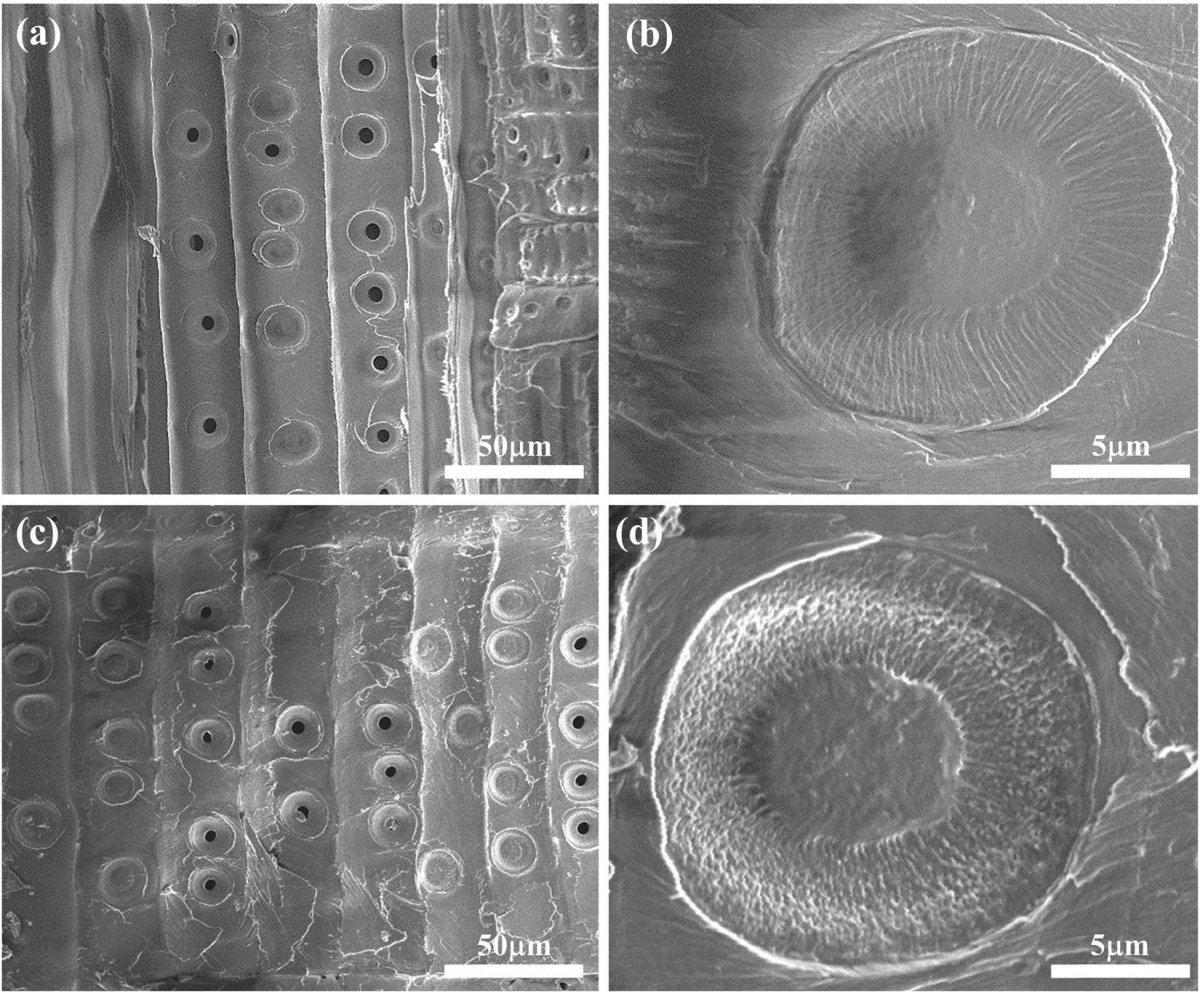
SEM images of radial sections of old spruce samples pretreated with 7.5% BMPDCA/ethanol solution. Samples in (**a**,**b**) were taken from an 18^th^-century buildings; (**c**,**d**) from an 18^th^-century French violin. The acceleration voltage is 5 kV.

In many archaeological studies, only minimal wood sampling is preferred. Researchers often need to work with extremely small wood samples (<10 mg), roughly cut using hand tools. They cannot be imaged satisfactorily using conventional SEM methods developed for bulk wood samples. By contrast, our IL pretreatment method worked very well with these samples. Figure 4 shows SEM images of bordered pits and pit membranes of some old spruce samples. These included specimens from old buildings (c. early 18th century) and an antique French violin (c. 1750). All of the results were the somewhat similar-the surface of the wood showed that a small fraction of the pits were unaspirated, but most of them were aspirated. The enlarged SEM images in Fig. 4(b,d) clearly reveal the torus and the margo. The torus is the thick, central part of the pit membrane, while the margo is the filamentous, surrounding material. Comparing the SEM images between these old spruce and modern spruce, the microstructures are similar. The attenuated total reflection-Fourier transform infrared spectroscopy (ATR-FT-IR) was also used to investigate the surface chemistry and chemical structures of spruces. The results in Fig. S5 reveal the chemical composition are similar for modern and antique spruces, except for the slight decrease of carbonyl signal in the old French violin around 1740 cm^−1^, caused by deacetylation of hemicellulose as a result of natural aging^24^ In previous reports^19,25^, clear visualization of torus/margo was only possible with conventional sample preparation but not with IL. In this study, however, we found an IL formulation that can achieve excellent imaging results without the time-consuming steps in conventional sample preparation method (slicing the surface by microtome, dehydration with a solvent, drying, mounting on a specimen holder and then applying metal coating).

**Figure 5:**
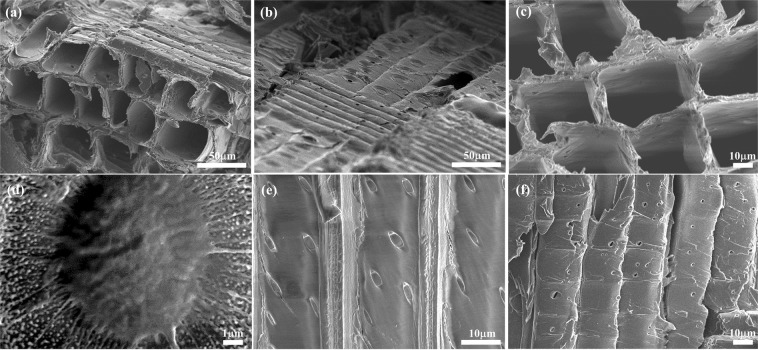
SEM images of a spruce sample from an18th-century French violin pretreated with 7.5% BMP-DCA/ethanol solution. (**a**–**c**) cross-section, (**d**) margo, and (**e**,**f**) radial section. The acceleration voltage is 5 kV.

**Figure 6:**
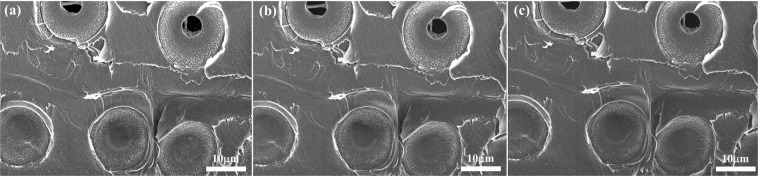
SEM images of 18th-century French violin spruce pretreated with 7.5% BMP-DCA and observed at different temperature (**a**) 60, (**b**) 90 and (**c**) 120 °C.

The cross-sectional views of the samples in Fig. 5(a–c) could be observed directly without any need for mounting or slicing. The samples were cut with ordinary scalpels into small pieces. The image in Fig. 5(a) shows many thin-walled tracheids dominating the image, and the radial sections of the spruce wood sample can be seen in Fig. 5(b). The different positions and perspective of the spruce wood are shown in Fig. 5(c–f). The excellent image quality of margo (Fig. 5(c)) and the radial section Fig. 5(d–f) indicate that the IL pretreatment method used in this study can replace the traditional pretreatment method. To acquire useful micrographs to observe the evolution of the microstructure during the heating is hardly possible because the image tends to drift and the spontaneous decomposition of the sample can result in breaking the vacuum as the temperature is increased. Due to the high thermal stability of the IL, favorable SEM images can be obtained at various temperatures, as shown in Fig. 6. The sample was first pretreated with a 7.5% of BMP-DCA solution, then placed on a specific heating stage with the temperature being adjusted from 30 to 120. The experiments were carried out under a high vacuum, and the images were recorded once the temperature had reached a stable value for five minutes. The micrograph is shown in Fig. 6(c) indicates that, even at an elevated temperature of 120°C, the structure of the sample remains the same, and a clear image is obtained. The experiment confirms that our pretreatment process not only allows the specimens to be maintained in their natural state for high-resolution imaging, even at high temperatures.

**Figure 7:**
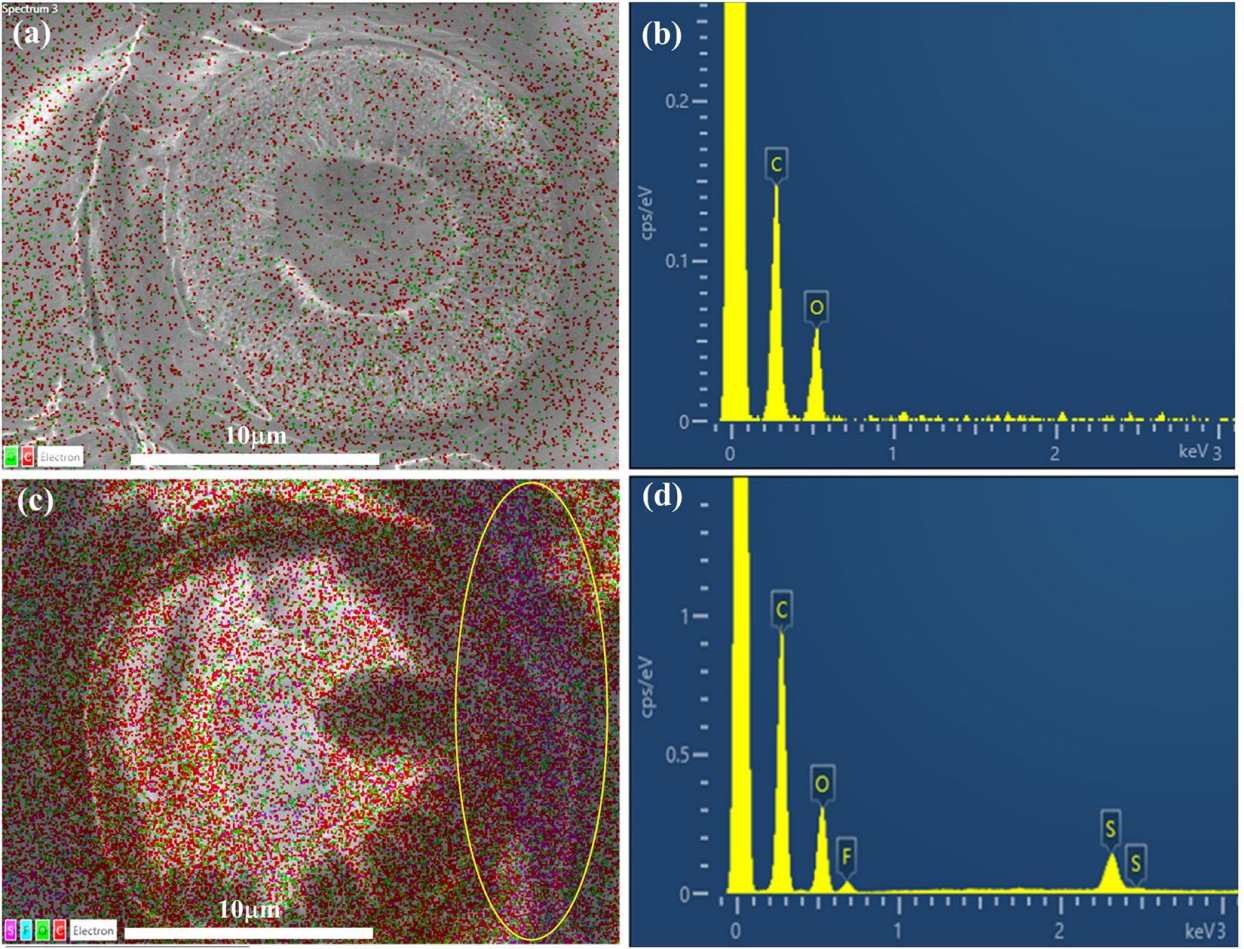
EDX mapping and analysis of a spruce sample from an 18^th^-century French violin pretreated with (**a**,**b**) 7.5% BMP-DCA and (**c**,**d**) 5% EMI-TFSI solution.

EDX mapping and analysis of a spruce sample from an old French violin that was treated with the BMP-DCA and EMI-TFSI solutions are shown in Fig. 7. Only carbon and oxygen could be detected, and they were well distributed on the bordered pit when the spruce was pretreated by the 7.5% of BMP-DCA solution, as shown in Fig. 7(a,b). No nitrogen from BMP-DCA was detected because the ionic liquid was spread out thinly over the substrate. On the contrary, sulfur, and fluorine can be detected in the EDX mapping when the 5% EMI-TFSI solution was used as the pretreatment reagent in Fig. 7(c,d). As shown in Fig. 7(c), the sulfur and fluorine were distributed on the right side of the pit membrane (circled area), because EMI-TFSI accumulated there. These results demonstrated that ILs do not necessarily cause problems with EDX elemental analysis, but care must be taken.

**Figure 8:**
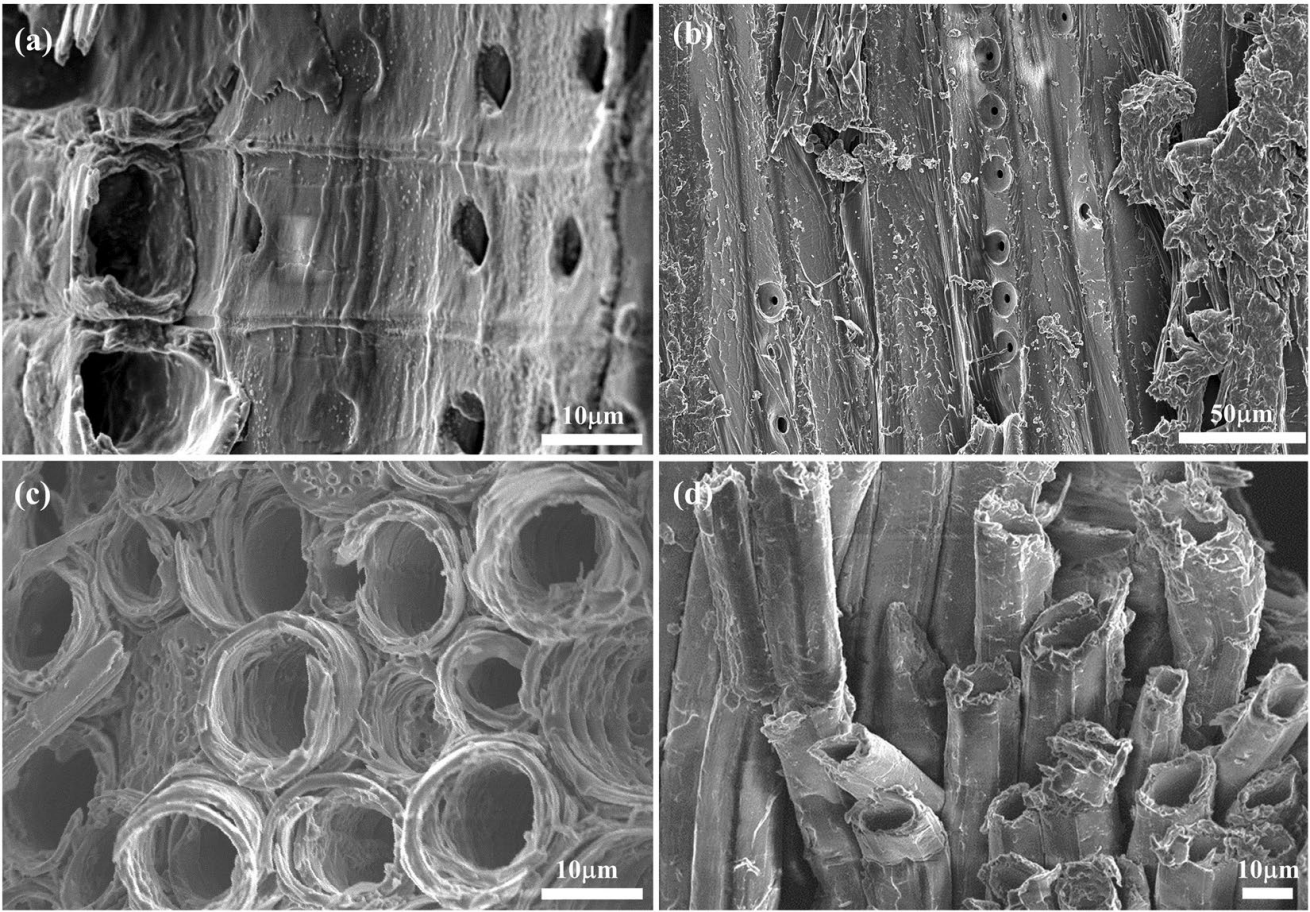
SEM images of various types of wood (**a**,**b**) fir spruce obtained from Chinese zither, and (**c**,**d**) maple wood obtained from 18^th^-century French violin.

To show that our pretreatment can be applied to a wide variety of antique wood samples, we tested Chinese fir wood from an antique Chinese zither (c. 9–10^th^ century) and maple from the back of an antique French violin (c. 1750). The former is a softwood species and the latter is a hardwood species, and both were satisfactorily imaged in Fig. 8. Even though the samples had rough surfaces compared to the spruce wood, the morphologies and structures were clearly visible with 7.5% BMP-DCA treatment.

## Conclusion

In summary, we were able to successfully obtain high-quality SEM images of wood samples by a simple and rapid pretreatment method. Four common ILs were tested as pretreatment reagents prior to the SEM examinations. The experimental results revealed that 7.5% BMP-DCA solution was optimal, likely due to its lower viscosity, lower density and considerable hydrophilicity. The hydrophilic IL also did not interfere with an EDX analysis compared to the hydrophobic IL such as EMI-TFSI. The spruce wood specimens still retained their structures without deformation and degradation, even at elevated temperatures. Our method is well suited for precious wood samples of archaeological nature. The amount of sample required is much smaller than that of conventional preparation method, and a thin and uniform IL conductive layer can be formed on the uneven surface, where metal sputtering often yields uneven coatings that compromise image quality. It worked well on both softwood and hardwood specimens taken from antique musical instruments, even up to 1000 years of age. Our method may become a useful tool for the routine and rapid SEM study of a wide variety of wood samples, including minute samples which cannot be easily handled by the conventional method.

## Methods

### Chemicals and reagents

We used four RTILs including 1-ethyl-3-methylimidazolium dicyanamide (EMI-DCA), 1-ethyl-3-methylimidazolium bis(trifluoromethylsulfony)imide (EMI-TFSI), 1- butyl-1- methylpyrrolidium dicyanamide (BMP-DCA), 1- butyl-1- methylpyrrolidium bis(trifluoromethylsulfony)imide (BMP-TFSI) in this study. The 1-ethyl-3-methylimidazolium chloride (EMIC, Fluka, 95%) was purified before use. 1-Methylpyrrolidine (Alfa, 98%), 1-chlorobutane (Alfa, 99%), Sodium dicyanamide (NaDCA, Alfa, 96%), and Lithium bis(trifluoromethylsulfony)imide (Li, TFSI, UR, 99%) were used as received. Those ILs were prepared and purified as described in a previous report^26^.

### Wood materials

Modern wood samples were generously provided by Sandro Chiao and Boa-Tsang Lee. An old French violin (c. 1750) was purchased from Mathias Renner. Spruce samples from an old building (early 18th century) were provided by Melvin Goldsmith. Chinese zither wood sample was generously provided by Kin Woon Tong.

### Measurements and instrumentation

The viscosity, density and water content of these ILs were measured by a cone/plate viscometer with a CPA40Z spindle attached (BrookField DV2T), a density meter (Anton Paar, DMA™ 35 Basic) and by Karl Fisher titrations using a Coulometer (Metrohm, 831 KF), respectively. Each spruce wood specimen was cut into a 0.5 cm* 0.5 cm square. Fig. 1 shows the pretreatment procedure using the RTILs. The spruce wood specimen was dipped into the IL solution for 30 s, and the excess IL was removed by the Kimwipes wipes. All of the samples were pre-vacuumed to remove ethanol before being introduced into the SEM chamber.

The surface structures and compositions of the wood specimens were characterized by scanning electron microscope (SEM, JEOL JSM-IT100) accompanied with a temperature controlling stage (Deben Peltier cool stage) and the energy dispersive X-ray analysis (EDX, Oxford Instruments).

Attenuated total reflection-Fourier transform infrared spectroscopy (ATR-FT-IR) was measured on Perkin Elmer Spectrum Two FT-IR spectrometer (Waltham, MA) and PIKE Technologies ATR accessory with ZnSe crystal (Fitchburg, WI). Wood samples were cut into small pieces and analyzed in absorbance mode with the following settings: wavenumber range of 650–4000 cm^−1^, resolution of 4 cm^−1^, and 32 scans for each spectrum. For each sample, 6–7 spectra were processed in the wavenumber range of 1187 to 1810 cm^−1^ by vector normalization and averaged by OPUS 7.5 software (Bruker, Billerica, MA). Vector normalization computes the average intensity of the selected wavenumber range, and the value is subtracted from the spectrum to center the mean. Subsequently, the spectra are scaled to make the sum squared deviation over the indicated wavelengths equal to one.

## Supplementary information

supplementary information
