# Supplementary material for: A facile ionic-liquid pretreatment method for the examination of archaeological wood by scanning electron microscopy
Source: Sci Rep. 2019 Sep 13;9:13253. doi: 10.1038/s41598-019-49773-y (PMC6744487; doi:10.1038/s41598-019-49773-y)
Supplement: Supplementary file 1 — supplementary information [file 41598_2019_49773_MOESM1_ESM.docx]

#### Supplementary Information

#### A facile ionic-liquid pretreatment method for the examination of archaeological wood by scanning electron microscopy

#### Bing-Jyun Lu^1^, Jia-Rong Li^1^, Hwan-Ching Tai^2^, Wenjie Cai^3,4^, Hsiao-Han Tseng^2^ and Yi-Ting Hsieh^1*^

^1^Department of Chemistry, Soochow University, Taipei City 11102, Taiwan

^2^ Department of Chemistry, National Taiwan University, Taipei 106, Taiwan

^3^ College of Arts and Landscape Architecture, Fujian Agriculture and Forestry University, Fuzhou, 350002, China

^4^ Graduate Institute of Creative Industries, College of Management, Shih Chien University, Taipei 104, Taiwan

^*^ythsieh@gm.scu.edu.tw


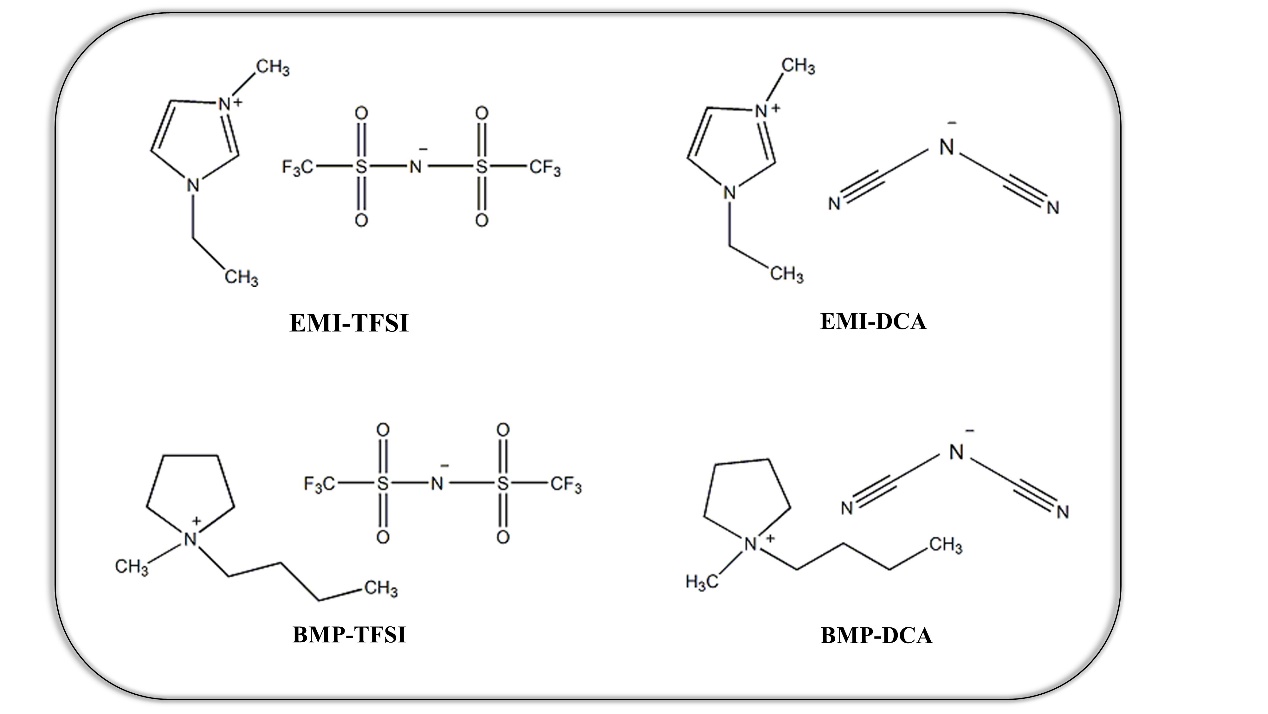


**Figure S1.** Chemical structures of ionic liquids used in this study.


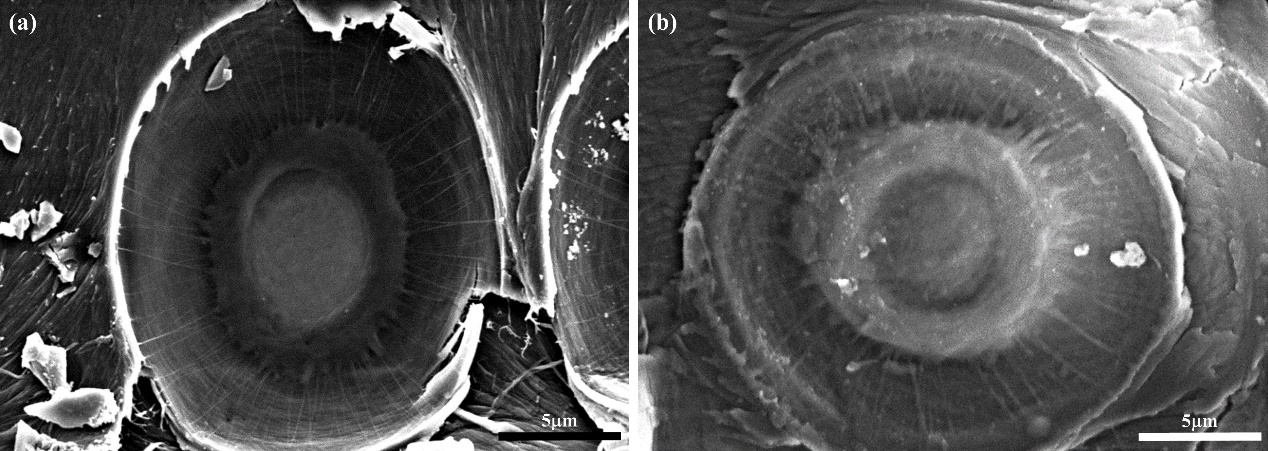


**Figure S2.** SEM images of modern spruce pretreated with (a) platinum sputtering and (b) 7.5 % BMP-DCA/ ethanol solutions. Acceleration voltage is 5 kV.


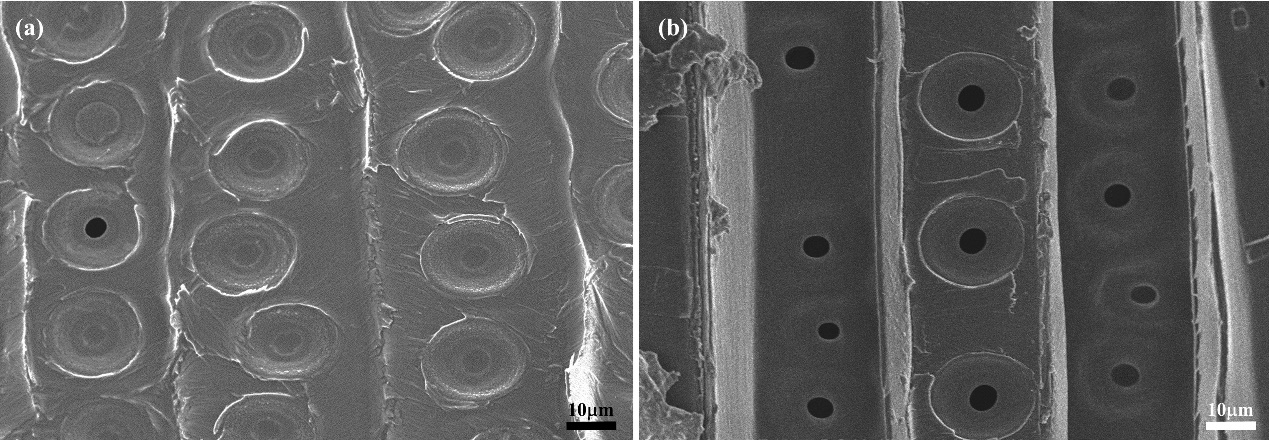


**Figure S3.** SEM images of modern spruce immersed in (a) neat BMP-DCA and (b) 7.5% BMP-DCA/ ethanol solutions for one month, then pretreated with 7.5 % BMP-DCA. Acceleration voltage is 5 kV.


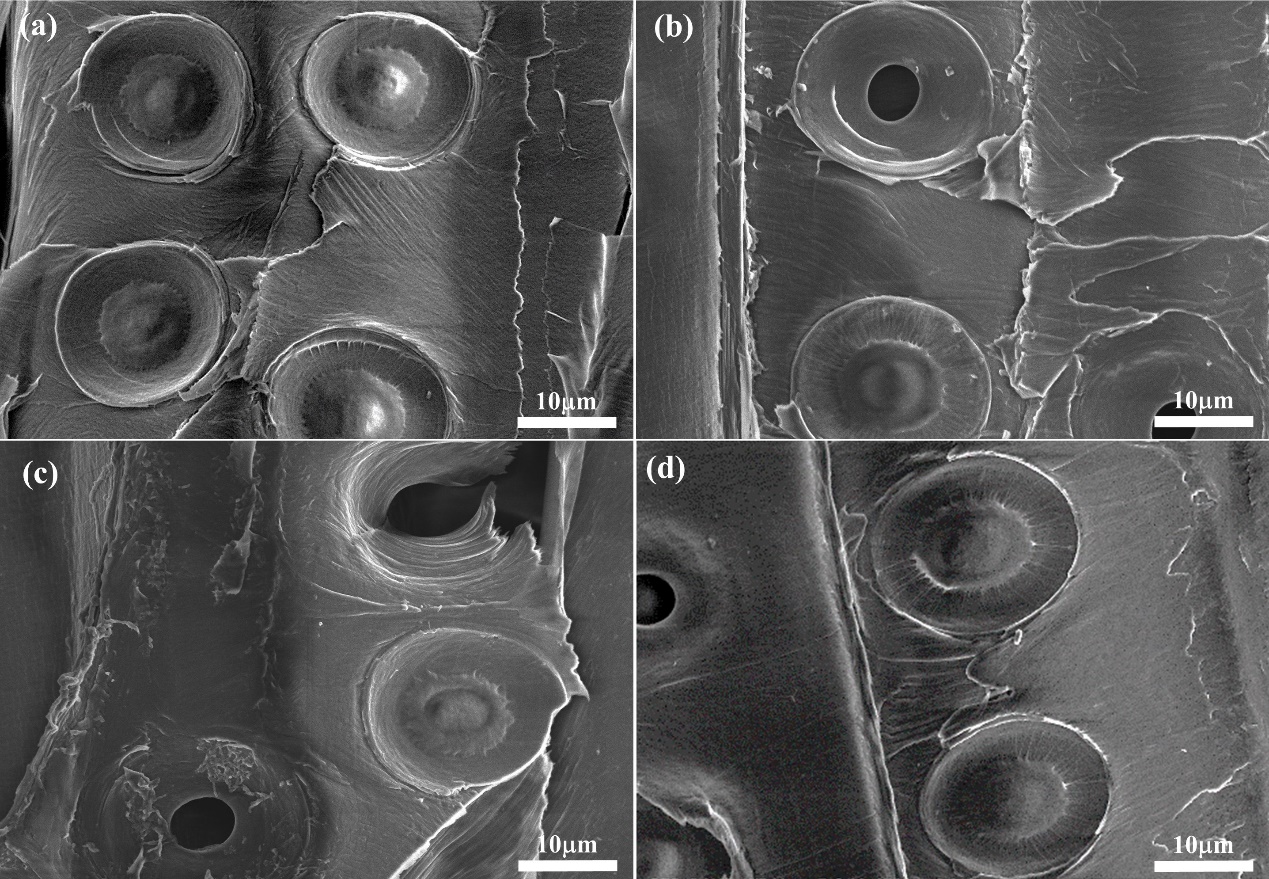


**Figure S4.** SEM images of modern spruce pretreated with 7.5 % BMP-DCA/ ethanol solutions for several times (a) first, (b) second, (c) third, and (d) fourth. Acceleration voltage is 5 kV.





**Figure S5**. Infrared absorption spectra of spruce wood.
